# Benefits and Risks of IgG Transplacental Transfer

**DOI:** 10.3390/diagnostics10080583

**Published:** 2020-08-12

**Authors:** Anca Marina Ciobanu, Andreea Elena Dumitru, Nicolae Gica, Radu Botezatu, Gheorghe Peltecu, Anca Maria Panaitescu

**Affiliations:** 1 Carol Davila University of Medicine and Pharmacy, Bucharest 020021, Romania; ciobanu.ancamarina@gmail.com (A.M.C.); gica.nicolae@umfcd.ro (N.G.); radu.botezatu@umfcd.ro (R.B.); gheorghe.peltecu@umfcd.ro (G.P.); 2 Filantropia Clinical Hospital, Bucharest 11171, Romania; helena_dumitru@yahoo.com

**Keywords:** immunoglobulin G, pregnancy, vaccine, transplacental transfer, autoimmune disorders, alloimmune disorders

## Abstract

Maternal passage of immunoglobulin G (IgG) is an important passive mechanism for protecting the infant while the neonatal immune system is still immature and ineffective. IgG is the only antibody class capable of crossing the histological layers of the placenta by attaching to the neonatal Fc receptor expressed at the level of syncytiotrophoblasts, and it offers protection against neonatal infectious pathogens. In pregnant women with autoimmune or alloimmune disorders, or in those requiring certain types of biological therapy, transplacental passage of abnormal antibodies may cause fetal or neonatal harm. In this review, we will discuss the physiological mechanisms and benefits of transplacental transfer of maternal antibodies as well as pathological maternal situations where this system is hijacked, potentially leading to adverse neonatal outcomes.

## 1. Introduction

Immunological adaptative changes occurring during pregnancy allow maternal tolerance towards the fetus and placenta, which practically constitute semi-allografts as 50% of their antigens have a paternal provenience [1]. This complex process mediated by maternal hormone levels and the placenta represents the fundament of a normally evolving pregnancy. Any breakdowns in this process may lead to maternal anti-fetal rejection and consequently to serious complications such as preterm birth, premature rupture of membranes, placental abruption or stillbirth [2]. During normal pregnancy, there is a physiological transfer of immunoglobulins from the mother to the fetus, with the aim of providing essential protection during the first few months of life when neonatal humoral response is inefficient.

The placenta has an extremely important function throughout gestation. It is a histological barrier formed by three layers: (1) multinucleated syncytiotrophoblasts (STB) with a cytotrophoblast layer directly beneath, (2) stroma containing fibroblasts and Hofbauer cells and (3) endothelial cells of the fetal capillaries [3]. There are active and passive mechanisms that allow transfer of low molecular weight substances from the mother to the fetus. Immunoglobulin G (IgG), although it has a high molecular weight, is the only class of immunoglobulins able to cross the placenta and to reach the fetal circulation.

IgG is a large protein with a tetrameric structure, containing two heavy and two light chains disposed in a Y-like shape. In its structure, there is an antigen binding site (Fab region) and a constant region, the crystallizable fragment (Fc), which binds to Fc receptors found on the surface of different cells in the immune system, most importantly on phagocytes. There are four types of IgG (1–4), and all of them are transferred across the placenta via syncytiotrophoblast cells that express receptors for the Fc domain, called neonatal Fc receptors (FcRn) [4,5,6].

Active transplacental transfer starts early in the course of pregnancy, at about 13 weeks of gestation, and fetal levels of IgG increase with advancing gestational age, being relatively low between 17 and 22 weeks at about 5–10% of maternal levels, then by 32 weeks reaching 50% of maternal levels, and finally exceeding maternal plasma IgG levels at birth [7]. 

Besides gestational age as an important factor in the transfer of antibodies, the process is influenced by several aspects such as maternal immunocompetence, concomitant infections, specific antibody levels acquired postimmunization, placental integrity, class of IgG and type of antigen [7]. These variables represent the basis for strategies regarding maternal immunization, aiming to protect newborns against infectious diseases, and also for the development of special surveillance protocols in situations such as maternal autoimmune or alloimmune conditions where harmful antibodies transferred across the placenta may cause severe fetal complications. 

## 2. Physiological Transfer of IgG

### 2.1. Role of FcRn

Neonatal Fc receptor is a major histocompatibility complex class I (MHC)-related molecule that plays a central role in the regulation of IgG homeostasis and transport across the placental barrier [8]. Expression of FcRn on syncytiotrophoblast cells appears to be decisive for efficient IgG-mediated phagocytosis [9], whereas expression on endothelial cells is important to prolong IgG half-life by recycling internalized IgG back to the surface [10]. 

**Figure 1:**
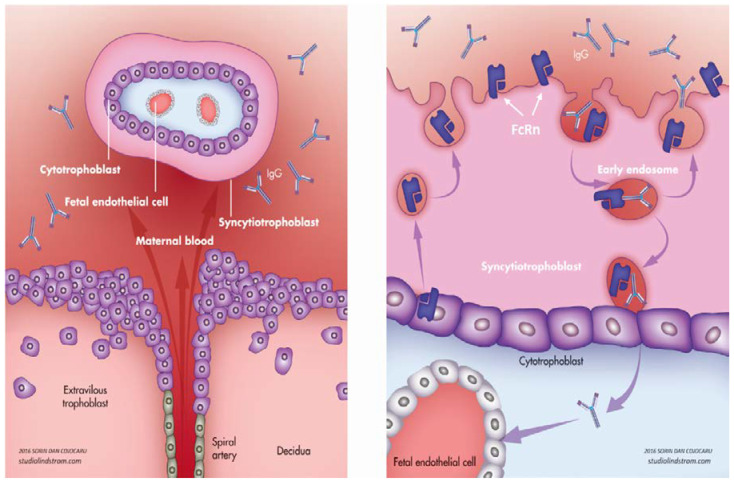
Transplacental passage of immunoglobulin G (IgG) and the neonatal Fc receptor. IgG from the maternal blood (**left**) is transferred by transcytosis at the level of syncytiotrophoblasts (STB); it is internalized within endosomes and binds to FcRn on the internal surface; the complex is then carried towards the basal cell membrane of STB and released into the fetal circulation (**right**). Purple arrows indicates IgG transport to the fetal blood.

The interaction between the Fc domain of IgG and FcRn is influenced by pH changes, presenting high affinity at acidic pH level < 6.5 and low affinity at physiologic pH [11]. During pregnancy, IgG is transferred from mother to fetus at the level of syncytiotrophoblasts through a process called transcytosis, which starts with internalization of maternal IgG into endosomes. At this level, the pH is acidified and it allows IgG to bind to FcRn present on the internal surface of the endosome [12]. The complex is then carried towards the basal cell membrane of the syncytiotrophoblast, where the IgG is released upon exposure to normal pH (7.4) and it enters the fetal circulation (Figure 1). It is not yet fully understood how maternal IgG is carried through villous stroma to the fetal endothelial cells as FcRn are not expressed on stromal cells [13,14]. Some evidence shows that Fc receptors type II and III are expressed in term placentas and could be involved in transplacental transfer of IgG [15]. It remains controversial whether FcRn are expressed or not on fetal endothelial cells, but Fc receptors II could be identified at this level and are considered to be linked with IgG passage into the fetal circulation [16,17]. 

In humans, FcRn are also expressed on both endothelial and bone marrow-derived cells, playing a role in prolonging the half-life of IgG and albumin [18,19] by preventing their catabolism. This finding might have a great impact in clinical practice: by modulating this IgG–FcRn interaction, one could improve the pharmacokinetics of therapeutic antibodies, or by inhibiting FcRn function in some autoimmune conditions, a reduced level of harmful antibodies could be achieved [20,21,22].

### 2.2. Timing of IgG Transfer and Other Influencing Factors

**Figure 2:**
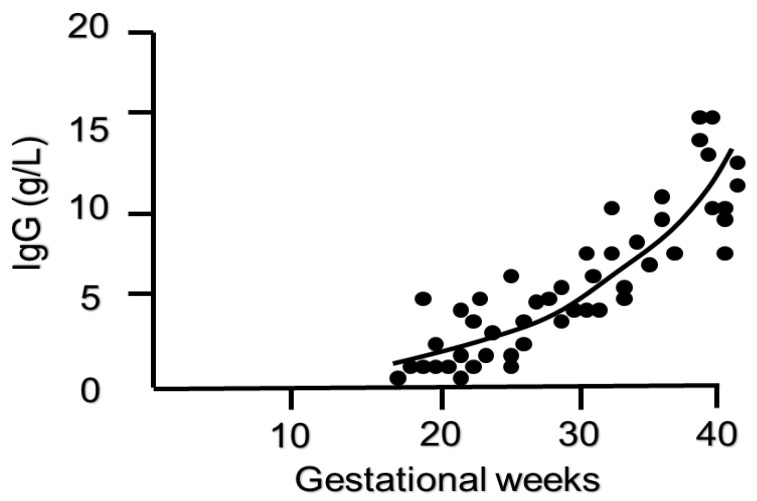
Timing of IgG transfer across the placenta in pregnancy (adapted from Malek A et al., Evolution of maternofetal transport of immunoglobulins during human pregnancy, Am J Reprod Immunol, 1996) [24].

The transplacental transfer of maternal antibodies to the fetus begins during the first trimester of pregnancy. Very little maternal IgG reaches the embryo and could be found in the coelomic fluid and villous stroma as early as 6 weeks [23]. Approximately 10% of maternal IgG concentrations are transferred to the fetus by 17–22 weeks’ gestation, and the levels show a continuous rise between 17 and 41 weeks, exceeding maternal levels by 37–40 weeks of gestation [24] (Figure 2). Due to this marked transfer of antibodies in the third trimester, closer to term, preterm neonates especially those born before 32 weeks have a considerably lower level of IgG compared to full term neonates and therefore higher susceptibility to infections [25,26,27]. Preterm infants also show a more rapid and earlier decrease in IgG in the postpartum period than term infants [28]. As passive immunization through transplacental maternal IgG transfer is not enough for preterm neonates, administration of intravenous immunoglobulin (iv IgG) after birth has been considered, although with limited benefits in reducing mortality or morbidity. A recent Cochrane meta-analysis tested the hypothesis that intravenous immunoglobulin supplementation in preterm or low birth weight neonates might reduce the risk of infections and any complications related to nosocomial infections. Data from more than 5000 infants enrolled showed that prophylactic use of iv IgG resulted in 3% reduction in sepsis and 4% reduction in severe infection, but without having a significant impact on neonatal mortality or other important neonatal outcomes such as length of hospital stay. Consequently, there is no general recommendation on prophylactic use of iv IgG in preterm neonates and there should be an individualized decision based on local resources. These results were consistent with previous data published in 2013 [29].

The immunoglobulin rise in the fetal circulation is different between the four types of IgG. The fastest transfer was observed for IgG1 and the slowest for IgG2. At term, in the fetal circulation IgG1 level was seven times higher than IgG2 level. There are also differences between the two IgG types compared to maternal levels: fetal IgG1 level exceed the maternal concentration near term or after birth, while fetal IgG2 remain below maternal level [24,30]. These differences were considered to be due to a preferential transfer of natural killer (NK) cell-activating antibodies, which could be explained through different affinities of neonatal Fc receptors towards specific components (glycans) in the Fc region [31].

Serum maternal immunoglobulin levels correlate to the placental transfer and neonatal IgG levels. Higher maternal IgG levels, above 15 g/L, have a negative influence on transplacental IgG passage and correlate to lower neonatal IgG levels, although in normal conditions fetal IgG levels in the third trimester surpass the maternal concentration. Paradoxically, when maternal level is too high, as is the case in maternal hypergammaglobulinemia, FcRn are oversaturated and unbound IgG molecules are destroyed [7], leading to decreased fetal antibody transfer. A similar effect is observed in black women, who normally have a higher level of total IgG than white women; therefore, one could say that transplacental transfer is correlated to maternal race [32].

On the other hand, in immunocompromised women secondary to HIV infection, reduced humoral immune response and reduced antibody production are linked with poor transfer of antibodies against specific pathogens such as group B streptoccocus, pertussis or Haemophilus influenzae [33,34]. In women with primary immunodeficiency with underlying specific gene mutation, exogenous IgG administration is essential to prevent severe maternal and fetal infections. It was observed that in these cases, immunoglobulin transfer across the placenta is similar to the transfer of endogenous antibodies [35]. 

With an unclear mechanism of impairment, placental malaria has been associated with reduced IgG transplacental transfer [34]. 

### 2.3. Maternal Immunization

#### 2.3.1. Vaccination

Pregnancy is associated with a particular background as the maternal immunological response is inhibited in order to tolerate the fetus and placenta. Some infectious diseases may be more severe during pregnancy, thus the most effective way to provide protection for the mother and the newborn is vaccination. Current guidelines recommend administration of inactivated vaccines, while live vaccines are contraindicated at least 4 weeks before conception and definitely during pregnancy. Vaccines routinely administered during pregnancy include inactivated influenza vaccine and Tdap, with some others such as those against pneumococcus, meningococcus, hepatitis A and hepatitis B being justified under specific circumstances [36]. 

Influenza vaccine in pregnancy was introduced in 2004 as part of a routine immunization program. It was observed that pregnant women are at greater risk of developing severe complications following influenza infection and that immunization in pregnancy, irrespective of trimester [37], reduces this risk and additionally provides protection for their newborns in the first few months of life [38]. 

A study on 112 mother–infant pairs highlights the strong correlation between maternal and neonatal antibody levels and the importance of passive immunity in newborns. As a consequence of low maternal immunity, only 30%, 42% and 82% of infants had protective antibody levels to pertussis, Haemophilus influenzae type b (Hib) and tetanus, respectively. Therefore, a high proportion of neonates are susceptible to those infections until active immunization is allowed [39]. In this context, Tdap (tetanus and diphtheria toxoids and acellular pertussis antigens) vaccination in pregnancy has proven its safety and significantly increases the titers of antibodies against those antigens. It is recommended from the second trimester, with best results when administered between 27 and 36 weeks of gestation and repeated in each pregnancy [40]. Administration in the third trimester provides the highest neonatal specific antibodies and prevents neonatal pertussis disease in the first 5–6 months of life until infants receive active immunization through vaccinations [41]. Enhanced placental transfer is influenced by maternal IgG levels, type of IgG and time between vaccination and delivery. It was shown that IgG type 1 preferentially crosses the placenta, compared to other types; therefore, polysaccharide conjugated vaccines such as group B streptococcus or pneumococcus conjugated to Tdap are able to preferentially induce maternal IgG type 1 production and to have a better impact on neonatal IgG levels [42].

It is important to mention that higher doses of passively acquired antibodies may suppress the immune response after vaccination in early infancy [43]. Several studies reported that maternal antibodies transferred across the placenta during pregnancy can decrease infant immunologic response to measles, tetanus and whole cell pertussis vaccines, with consequent production of lower levels of antibodies postimmunization [44,45,46]. Despite this disadvantage, the benefits for newborns in the first few months of life in terms of reducing hospitalization, decreasing the risk of mechanical ventilation and even decreasing the risk of death support the current recommendation of maternal Tdap immunization in the third trimester of pregnancy. 

Due to general vaccination programs, most data showed high seropositivity for measles, mumps, rubella and varicella-zoster (VZV) in women of childbearing age. Maternal antibodies and transplacental passage protect newborns in the first few months of life against most vaccine-preventable diseases, and this protective level is maintained up to 6 months of age. In normal conditions, the rubella, measles or VZV antibody titer in the cord blood of neonates born at term exceeds maternal antibody levels. There is a high prevalence of anti-rubella and anti-VZV protective IgG titers, but there is a decreasing trend in anti-measles and anti-mumps immunity levels in pregnant women and neonates. Therefore, new preventive strategies have been proposed, such as measles-mumps-rubella (MMR) screening and vaccination of susceptible women of childbearing age or MMR vaccination of women who have no documentation of completed vaccination and no laboratory evidence of immunity [47,48,49]. Live vaccines such as MMR and varicella vaccines are contraindicated during pregnancy, and maternal serologic status should be checked before conception in order to prevent any possible infections during pregnancy by appropriate preconceptional immunization [50].

Passively acquired maternal antibodies with different antigen-specificities have been found to have different half-lives in infants. For example, although in normal pregnancy pertussis-specific IgG levels in cord blood achieve more than 100% of maternal levels, maternal pertussis-specific IgG has a half-life of 6 weeks in infants and decreases to undetectable levels as early as 4 months of life [51]. In contrast, maternal passively acquired measles-specific IgG remains near protective levels at 6 months after birth and is still detectable at 1 year of life. 

Ongoing efforts are being made to develop future vaccines for maternal immunization, the two most promising vaccines being respiratory syncytial virus vaccine, in phase III trial, and group B streptococcus vaccine to prevent late-onset neonatal infection, currently in the early stages of development. A vaccine against cytomegalovirus is also under development [36,52,53,54]. 

New emerging viruses with potential harm for the fetus, such as the Zika and Ebola viruses, have also been under investigation for the development of future vaccines [55,56]. 

#### 2.3.2. Maternal Infection with COVID-19

The greatest challenge of our time is the COVID-19 pandemic, and special attention has been drawn to pregnant women and their infants in terms of vertical transmission, severity of the disease and immunologic response. Although there is no clear evidence of vertical transmission due to lack of angiotensin-converting enzyme 2 (ACE2) receptors for SARS-CoV-2 virus in the placenta [57], the immunologic status at birth was investigated in 71 neonates born to mothers with confirmed infection. Immunoglobulin M (IgM) does not cross the placenta; it is a result of fetal immunologic response to pathogens, an ability that is acquired early in the first trimester of pregnancy. IgG in the fetal blood at birth is a result of maternal immunoglobulin transferred across the placenta. In 20% of the investigated cases, IgM was detectable after birth and in one case it was positive at 2 h postpartum, but the PCR remained negative after multiple determinations and we should take into account cross-reactivity and false positive rate of IgM results before making any assumptions [58].

In a small cohort of six infants born to mothers with COVID-19 infection, IgG was present in all cases and the neonatal level was well correlated with maternal immunoglobulin levels. However, the study did not investigate whether the presence of antibodies in the neonates had a protective effect against infection as there was no long-term neonatal antibody level follow-up [59]. One case report showed that IgG to SARS-CoV-2 in the neonate decreased in less than one and a half months after delivery, suggesting the potential risk for subsequent COVID-19 in neonates. The presence of IgG and immunoglobulin A (IgA) antibodies was confirmed in breast milk, indicating that breastfeeding might have an important immune protection for infants after birth [60,61]. Current recommendations state that for assessing future vaccine efficacy, maternal vaccination should be considered early in the design of these trials [62]. 

## 3. Pathological Transfer of IgG—Autoimmune Disorders

Transplacental immunoglobulin transfer plays an important role in protecting the newborn in the first few months of life, when the immune system is immature and active immunization through vaccination is not yet recommended. Despite this clear benefit, there are situations in which transplacental transfer may be damaging to the fetus. Particular maternal conditions are further discussed.

### 3.1. Graves’ Disease—TRAb Antibodies

Fetal or neonatal goiter may be the result of transplacental passage of maternal antibodies in women with Graves’ disease and occurs in almost 20% of cases [63]. Antibodies that modulate the thyroid-stimulating hormone (TSH) receptor are the hallmark of Graves’ disease, and increased maternal levels, three times above the normal limit, can cross the placenta into the fetal circulation and cause fetal goiter, frequently associated with fetal hyperthyroidism [64]. Acting on the TSH receptor, thyroid receptor antibodies (TRAb) have different effects: they can stimulate (TSAb), block (TBAb) or exert a neutral effect (N-TRAb). It should also be mentioned that maternal anti-thyroid medication given to control maternal hormonal levels or, very rarely, thyroid-blocking antibodies (TBAb) could cross the placenta and cause fetal hypothyroid goiter. Differentiation of the etiological mechanism involved in fetal goiter is important for adequate treatment selection, and it can be achieved by clinically assessing the situation of the mother or by performing fetal blood sampling to detect TSH and free T4 levels in the umbilical cord. 

Due to pregnancy-induced immunosuppression, TRAb levels detectable in the first trimester tend to decrease after 20 weeks of pregnancy, resulting in the amelioration of the thyrotoxicosis in women with Graves’ disease. TRAbs can cross the placenta from the first few weeks of pregnancy, and the rate increases with advancing gestation. Thus, it is recommended to measure maternal TRAb concentrations in the first trimester and at 24–28 weeks of gestation and to monitor the pregnancy more carefully if antibody levels surpass three times the normal values. 

**Figure 3:**
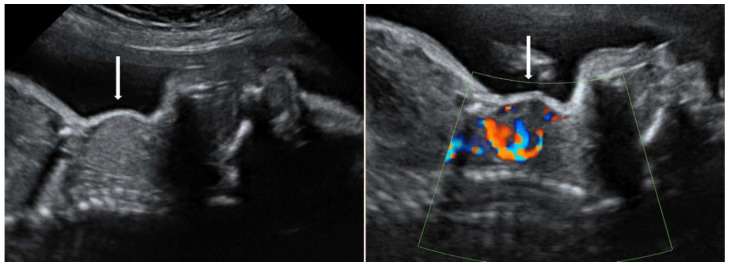
Fetal thyroid goiter (arrow) in gray-scale and power Doppler (courtesy of the Fetal Medicine Foundation).

Ultrasound evaluation along with maternal history and TRAb levels are important to establish the diagnosis. The fetal thyroid gland can be assessed by ultrasonography after 20 weeks of gestation, when fetal goiter appears as an anterior cervical echogenic mass (Figure 3). Depending on its size, fetal goiter can be associated with other complications such as compression of the esophagus or trachea at birth, impeding swallowing and consequently leading to polyhydramnios [65]. Fetal hyperthyroidism can cause fetal growth restriction (FGR), accelerated bone maturation and craniosynostosis, tachycardia, cardiac failure and hydrops. Fetal hypothyroidism is associated with decreased bone maturation, normal heart rate, reduced fetal movements and long-term neurological sequelae. If ultrasound signs cannot clearly differentiate between the two types of fetal goiter, amniocentesis or cordocentesis might be used to determine fetal thyroid status [66,67].

### 3.2. Systemic Lupus Erythematosus, Sjögren’s Syndrome—Anti-Ro, Anti-La Antibodies

Anti-Ro and anti-LA antibodies were initially described in association with systemic lupus erythematosus (SLE) and Sjögren’s syndrome, but they can also be found in relation to other autoimmune diseases such as mixed connective tissue disease or systemic sclerosis [68]. Some patients are completely asymptomatic, around 3% of the general population, and the presence of antibodies in this group of patients is usually diagnosed upon fetal complications. 

Neonatal lupus is a passively acquired autoimmune disorder as a result of transplacental passage of maternal anti-Ro/anti-La antibodies, and this condition can have cutaneous, hematologic or cardiac manifestations. The most threatening complication is fetal complete atrioventricular block, which occurs in 2% of nulliparous women with positive anti-Ro antibodies and increases to 20% in pregnancies with a previously affected fetus [65,69]. Anti-Ro antibodies are directed against two different proteins called autoantigens Ro-52 and Ro-60 according to their molecular weight. The presence of each antibody and its clinical implications in the development of congenital heart block (CHB) have been the subject of different studies. Some authors suggested that anti-Ro 52 antibodies have a predominant role in CHB, especially the anti-p200 antibodies, which are a subclass of anti-Ro 52 antibodies directed against a specific region of the Ro-52 protein. However, the frequency of anti-Ro 60 antibodies in association with CHB varies depending on the immunoassay method. Gordon et al. showed that both types of antibodies are equally involved in the pathogenesis of CHB. A recent study on 144 pregnant women showed that a low titer of isolated anti-Ro 60 antibodies is associated with a favorable pregnancy outcome, and this finding could possibly change the frequency of monitoring by fetal echocardiography [70,71,72,73]. The risk of CHB is also related to maternal antibody levels. If the maternal antibody titer is >50 UI/mL, the risk of developing CHB is as high as 5% [74].

Prenatal counseling is important because neonatal lupus is responsible for 80 to 95% of all cases of congenital complete heart block in the absence of structural defects diagnosed in utero or in the neonatal period [75,76]. Congenital heart block is thought to result when anti-Ro and anti-La antibodies bind to fetal conductive cardiac cells, generating an inflammatory injury and fibrosis of the atrioventricular node [77]. Transplacental transfer of these antibodies starts in the early second trimester, and the greatest risk for CHB is between 16 and 28 weeks’ gestation.

Third-degree heart block can lead to heart failure and to hydrops. The risk of death amongst affected fetuses is about 15%, and 70–80% of the survivors require placement of a pacemaker within the first 10 years of life [78,79].

Due to increased mortality and morbidity, in pregnant women with known positive immune status, close monitoring should be carried out by a multidisciplinary team during pregnancy and immediately after birth [80]. As a standard method of screening and diagnosis of CHB, some recommend to perform weekly pulse Doppler fetal echocardiography starting at 16 weeks of gestation, with measurement of mechanical PR interval, considered an equivalent of the PR interval on the electrocardiogram, although there is still controversy about its predictive value in cases that will evolve from first-degree heart block to complete atrioventricular block [81,82].

Regarding the skin manifestations, the typical rash with annular lesions mainly located on the scalp or periorbital area with a raccoon-like appearance can be present at birth or may not develop until exposure to ultraviolet lamp. The rash is self-limiting, resolving in approximately six to eight months due to the half-life of IgG of about 21–25 days [83,84].

The risk of having a child with complete heart block increases with the titer of maternal antibodies and maternal history of previous affected pregnancies.

### 3.3. Primary Autoimmune Thrombocytopenia—ITP Antibodies

Another autoimmune maternal condition that may affect the management of pregnancy and fetal outcome is the immune-mediated thrombocytopenia. Primary autoimmune thrombocytopenia (ITP) in pregnancy has an incidence that ranges between 1 in 1000 and 1 in 10,000 pregnancies [85,86]. Compared to gestational thrombocytopenia it is less frequent, but it is considered the main cause of isolated thrombocytopenia in the first and second trimesters of pregnancy, while gestational thrombocytopenia is encountered later in gestation with a milder platelet decrease. Its pathological mechanism consists of accelerated destruction of platelets, mainly in the spleen, due to antibodies directed against platelet membrane glycoprotein complexes [87]. The transplacental transfer of IgG platelet-specific autoantibodies can induce fetal platelet destruction and cause neonatal thrombocytopenia. 

The majority of infants born to mothers with ITP may have normal or mild thrombocytopenia, but the full count may decrease sharply in two to five days after birth [88]. The main concern is the increased risk of intracranial hemorrhage (ICH) that can manifest itself from the fetal life. Although there is no direct correlation between fetal/neonatal and maternal platelet counts, the risks of maternal and neonatal morbidity and mortality are highest in women with more severe forms of disease [89]. Paraclinical investigations such as fetal scalp blood count or cordocentesis can predict the amount of neonatal platelets, but these invasive methods are rarely used in practice since they can increase the risk of bleeding. Pregnancy management, treatment and mode of delivery are dictated by maternal platelet count. Treatment options and indications for pregnant women are similar to those recommended for adult ITP patients, but it is important that at the time of delivery, the minimum platelet count is >80 × 10^9^/L for epidural anesthesia and >50 × 10^9^/L for cesarean delivery [90].

Prenatal or preconceptional screening for ITP is recommended, especially when the obstetric history shows an increased risk, for example a previously affected child. 

### 3.4. Myasthenia Gravis—Anti-AChR Antibodies

**Figure 4:**
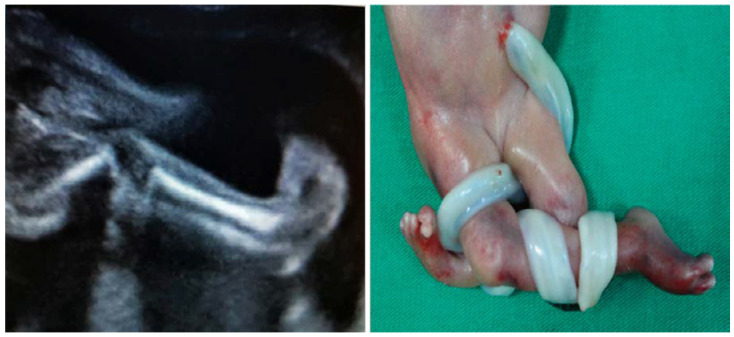
Fetal arthrogryposis with talipes (collection of Filantropia Hospital).

Myasthenia gravis (MG) is another autoimmune disease that may interfere with the development of a normal pregnancy and neonatal outcome. This neuromuscular disease is characterized by autoantibodies directed against the acetylcholine receptors (AChR) found at the neuromuscular junction of the skeletal muscles. Myasthenia gravis affects between 1 in 10,000 and 1 in 30,000 pregnant women, depending on the geographic area [91], and transplacental passage of AChR antibodies or anti-MuSK (anti-muscle-specific kinase) from the affected mother to the fetus can produce transient neonatal myasthenia gravis in 15% [92] of cases or a more severe condition, arthrogryposis multiplex congenita, in about 2% of cases [93]. Two types of autoantibodies against Ach receptors have been described: the adult type, which seem to be involved in maternal and transient neonatal MG, and the fetal type, which are found in the fetal neuromuscular junction up to 33–35 weeks of gestation and may be more frequently involved in fetal arthrogryposis congenita [94]. This condition can be prenatally diagnosed by ultrasound, and it is characterized by polyhydramnios, lack of movement and abnormal position with fixed flexion or extension deformities in fetal joints [95] (Figure 4).

The severity and duration of maternal MG does not correlate with the risk of transient neonatal disease, but the history of a previous affected child can predict a risk of recurrence of about 75%.

### 3.5. Autoimmune Hemolytic Anemia—wAIHA Antibodies

Autoimmune hemolytic anemia (AIHA), primarily caused by pregnancy, is characterized by erythrocyte autosensitization and development of antibodies against one’s own red blood cell antigens. Autoimmune hemolysis associated with pregnancy occurs very rarely, with an incidence estimated at 1 in 50,000 pregnancies [96]. 

Autoimmune hemolytic anemia may be secondary to warm, cold, or a mix of warm and cold autoantibodies [97]. Warm-active antibodies refer to IgG antibodies that maximally bind red blood cells at body temperature and represent the cause of autoimmune hemolytic anemia in 80–90% of cases [98]. It is important to make the correct differential diagnosis with other causes of hemolytic anemia in pregnancy such as HELLP syndrome, acute fatty liver of pregnancy, thrombotic thrombocytopenic purpura or hemolytic uremic syndrome as different management strategies are required [99].

Maternal warm (IgG) autoantibodies can cross the placenta and determine variable forms of hemolytic disease in the fetus, ranging from mild disease to severe hemolysis. The risks to the infant were increased when erythrocyte autoantibodies were part of other active autoimmune conditions such as systemic lupus erythematosus. Evans syndrome, which is associated with autoimmune hemolytic anemia and immune thrombocytopenic purpura, carries high risks of fetal morbidity, including severe hemolysis and intracranial bleeding with neurological sequelae and death [100]. 

**Figure 5:**
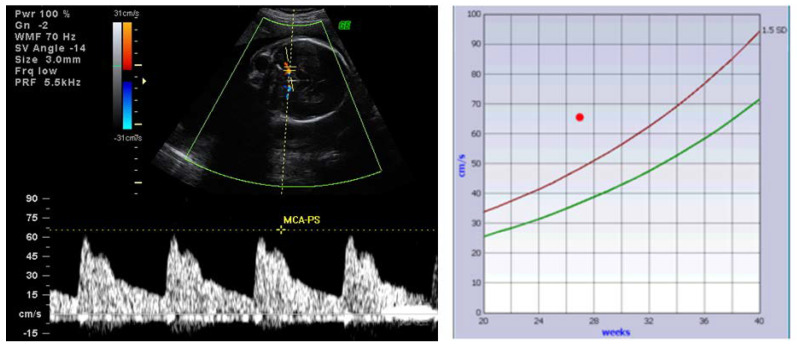
Pulsed Doppler examination showing increased peak systolic velocity on the middle cerebral artery (**left**); When plotted on the graph (**right**), the peak systolic velocity on the middle cerebral artery in this case (red dot) is higher than the limit of 1.5 standard deviation (red line); the green line represents the median peak systolic velocity for gestational age (collection of Filantropia Hospital).

Close fetal surveillance with Doppler examination of peak systolic velocity on the middle cerebral artery can predict cases at increased risk of fetal complications and dictate the appropriate management [101] (Figure 5).

### 3.6. Autoimmune Bullous Diseases

Neonatal autoimmune blistering disease (AIBD) has a low incidence, and only a few case reports have been published so far. Neonates born to mothers who are known before conception to have autoimmune blistering diseases such as pemphigus vulgaris (PV), pemphigus foliaceus (PF), bullous pemphigoid (BP), linear Immunoglobulin A bullous dermatosis (LABD) or epidermolysis bullosa acquisita (EBA) may be affected due to transplacental passage of IgG autoantibodies. Usually these are transient conditions and improve spontaneously within a few weeks after birth, after maternal autoantibodies gradually decrease in the neonate’s blood. Autoimmune blistering disease might increase the risk of preterm birth and growth restriction, and there have also been reported cases of stillbirth and neonatal death related to pemphigus vulgaris. In the case of neonatal blistering disease, specific investigations should be undertaken in order to confirm the etiology. Confirmation of the autoantibodies involved is essential. Autoantibodies anti-desmoglein 1 and 3 (Dsg1,3) are related to PV or PF (only anti-Dsg-3), anti-BP180 antibodies are related to bullous pemphigoid, and anti-type VII collagen antibodies are found in EBA. Cases of neonatal LABD were independent of maternal disease and appeared to have a more severe blistering form [102,103].

**Figure 6:**
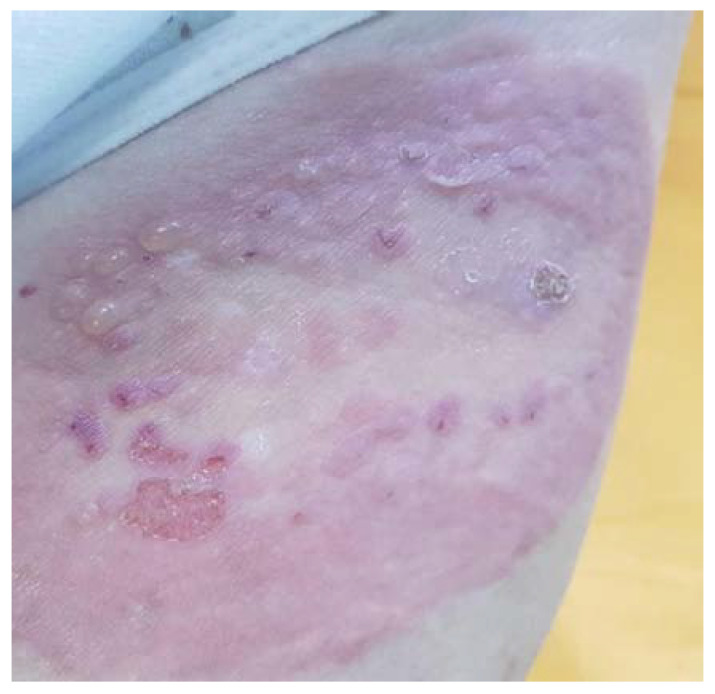
Pemphigoid gestationis: cutaneous bullous lesions on the upper thigh of a third trimester pregnant patient (courtesy of Prof. Călin Giurcăneanu).

A rare autoimmune disease associated with pregnancy is pemphigoid gestationis (PG), previously known as herpes gestationis. This is an AIBD caused by the production of autoantibodies against adhesion molecules, mainly against bullous pemphigoid BP180 or collagen XVII [104]. Clinically the condition is described as itchy, red skin with blisters mainly in the umbilical zone, but also in other areas of the body (Figure 6) appearing in the second and third trimesters [105,106].

As a consequence of transplacental passage, neonatal PG can develop in 10% of cases. This is a mild condition that resolves spontaneously as antibody levels decrease. Another important implication is that BP180 antibodies can also attack chorionic cells with detachment of basement membranes and undeveloped hemidesmosomes, resulting in placental insufficiency associated with fetal growth restriction, preeclampsia or premature delivery [104,107].

There is little knowledge about the subject, but close fetal monitoring is recommended in the case of maternal symptoms due to risks for prematurity and fetal growth restriction [107].

## 4. Pathological Transfer of IgG—Alloimmune Disorders

Around 50% of fetal antigens have a paternal origin, and maternal immunization to different fetal antigens inherited from the father, unrecognized as ″self″, determines an immune response with production of antibodies and placental transfer into fetal circulation leading to destruction of different fetal cells, mainly blood cells.

### 4.1. Alloimmune Hemolytic Disease of the Fetus and Newborn (HDFN)—Anti-Red Cell Antibodies

Human red cells express hundreds of different blood group antigens, and some of them have been reported to determine a maternal alloimmune response. 

The Rh system is the most complex of the human blood group systems, consisting of about 45 antigens. Clinically, the most important antigen is D. This antigen is immunogenic and is well developed early in gestation; 12% of the Caucasian population is D-negative, and the maternal antibodies cause fetal hemolysis [108]. Small volumes of fetal red cells enter the maternal circulation during most normal pregnancies and after most normal deliveries. In the absence of Rh prophylaxis, about 16% of D-negative women carrying their first D-positive pregnancy become immunized. In subsequent pregnancies, anti-D antibodies can be transferred across the placenta and if the fetus inherits D antigen from the father, it is at great risk of hemolytic anemia. All antibodies to Rh-system antigens should be considered capable of causing severe HDFN, but the most frequent ones are anti-D and anti-C antibodies. 

HDFN in the ABO system is restricted almost exclusively to the fetuses of group O mothers. ABO HDFN requiring any clinical intervention before birth is rare as A and B red cell antigens are not fully developed in the fetus. Also, A and B antigens are histo-blood antigens that can be found in other cells, for example in the placenta; therefore, antibodies transferred from the mother are held and destroyed at this level. 

In the context of generalized screening and prophylaxis against anti-D immunization, another antigen, part of the Kell blood group system, has attracted attention in the etiology of HDFN. The K-positive phenotype is present in about 9% of Europeans, and the anti-K antibody is considered the most common immune red cell antibody outside of the ABO and Rh systems [109]. In immunized pregnancies, its presence causes severe fetal anemia due to both hemolysis and suppression of erythropoiesis [110].

### 4.2. Alloimmune Thrombocytopenia—Anti-HPA Antibodies

Alloimmune thrombocytopenia is found in about 1 in 2000 pregnancies [111] and in most cases is caused by production of maternal antibodies against human platelet antigen-1a (HPA-1a) or HPA-5b found on the fetal platelets’ surface and inherited from the father [112]. About 2.5% of pregnant Caucasian women are HPA-1a-negative, but only about 10% of HPA-1a-negative women will develop anti-HPA-1a antibodies [113].

Unlike HDFN, in alloimmune thrombocytopenia, maternal sensitization to fetal platelet antigens often occurs in the first pregnancy, indicating that platelet antigens may be more immunogenic than red cell antigens. In alloimmune thrombocytopenia, compared to autoimmune maternal conditions, the severity of fetal platelet decrease is greater and consequently the risk of intracranial bleeding is higher, estimated at around 20% [111]. Also, the risk of recurrence in future pregnancies is high, at 50–100% depending on paternal heterozygosity or homozygosity antigen status [65]. 

**Figure 7:**
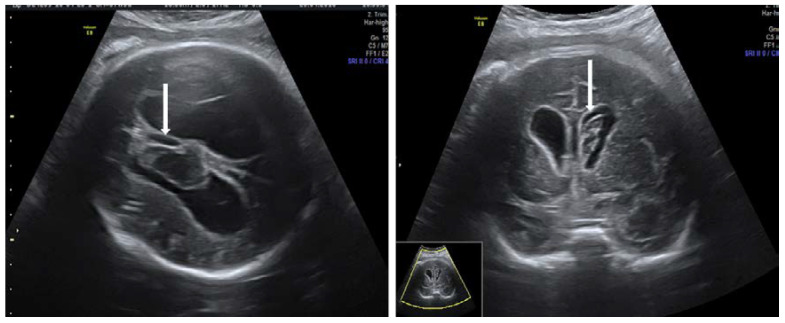
Fetal ventriculomegaly due to intraventricular hemorrhage (arrow) (collection of Filantropia Hospital).

Unfortunately, as currently there is no routine screening for HPA antibodies, most cases are diagnosed after birth or when severe fetal thrombocytopenia leads to intracranial bleeding that could be detected by ultrasound evaluation. Ultrasound findings in the case of intracranial hemorrhage are ventriculomegaly, clots within lateral ventricles, porencephaly, signs of severe anemia and hydrops [65] (Figure 7). Due to increased risk of recurrence, active management initiated early in subsequent pregnancies might reduce the risks of morbidity and mortality from severe hemorrhage [114,115,116].

### 4.3. Gestational Alloimmune Liver Disease, Neonatal Hemochromatosis—Anti-Hepatocyte Antibodies

Gestational alloimmune liver disease (GALD) is an alloimmune disorder and the main cause of neonatal acute liver failure. The disease is caused by maternal IgG directed against fetal hepatocytes, and although the exact antigen is not yet described, it is supposed to be expressed early in embryonic life on the hepatocytes [117]. Another theory supports that the etiology of GALD is not related to proteins inherited from the father and absent in the mother, but that actually women may be congenitally deficient in some protein normally expressed on hepatocytes and therefore develop alloantibodies [118]. The clinical implications are expressed at birth by cirrhosis, severe liver failure and extrahepatic iron accumulation, mainly in the pancreas, heart and thymus, a condition described as neonatal hemochromatosis. Extrahepatic siderosis is the consequence of liver disfunction in regulating the iron flux from the placenta to the fetus. In GALD, hepcidin, the feedback molecule that inhibits ferroportin function, is reduced, which leads to abnormal iron accumulation [119].

GALD has a poor prognosis associated with a high risk of mortality, close to 80%, and need for liver transplantation. A woman may have several unaffected pregnancies but once the condition occurs, the risk of recurrence in subsequent pregnancies is about 90%. Correct diagnosis and appropriate management initiated early in pregnancy might prevent recurrence in almost 90% of cases [120,121].

Different clinical implications in both the mother and the fetus are presented in Table 1, comparing autoimmune and alloimmune disorders.

## 5. Pathological Transfer of IgG—Biological Therapy in Pregnancy

Numerous autoimmune disorders have been identified so far, and almost 80% are encountered in women of childbearing age [122]. The evolution of autoimmune disorders is variable during pregnancy. In some situations, the disease is triggered by pregnancy or the postpartum period; some conditions relapse or are aggravated during gestation; some autoimmune disorders ameliorate under the immunosuppressive influence of pregnancy. For example, rheumatoid arthritis or systemic sclerosis might improve during pregnancy while systemic lupus erythematosus, myasthenia gravis or inflammatory bowel disease might present a worse evolution with gestation [123,124,125,126]. For a better outcome, it is recommended to plan conception when the disease is in remission or well controlled, and recent evidence indicates that treatment should be adapted and continued during pregnancy in order to minimize the risk for the mother and the fetus. 

Anti-tumor necrosis factor alpha (anti-TNFα) antibodies are probably the most studied biological therapy used in pregnancy. Anti-TNFα antibodies such as infliximab and adalimumab are IgG1 antibodies that have a preferential transfer across the placenta starting at the end of the second trimester; therefore, neonatal drug levels may exceed maternal levels and may persist up to 12 months after birth [127]. Etanercept, a fusion protein with a modified Fc portion that binds to TNFα, has a low capacity to cross the placenta, and minimal levels are found in the fetus at birth. Certolizumab, an anti-TNF therapy missing the Fc portion, does not cross the placenta and can be used throughout pregnancy. 

The current data show that pregnancies exposed to anti-TNFα are not associated with an increased risk of fetal malformations, preterm delivery or pregnancy loss [128,129,130]. One of the main concerns, when the fetus is exposed in utero to biological therapy, is the risk of neonatal infections, particularly in the first year of life [131]. The observational data indicate that infants exposed to anti-TNF agents are not at greater risk of severe infections compared to the unexposed population, unless thiopurine therapy (azathioprine) is involved. In this case, the risk can be three times higher [128]. Current recommendations regarding the use of biologics during pregnancy advise the discontinuation of medication at 20 weeks of gestation for infliximab and adalimumab and around 32 weeks for etanercept [132]. If the medication is continued beyond 28 weeks, the drug levels in the fetus will exceed those in the mother, and precautions and close monitoring are required after birth. A large database of 1457 pregnant women with inflammatory bowel disease receiving anti-TNFα medication, mainly infliximab and adalimumab, showed that discontinuation of treatment before week 24 increased the risk of maternal disease flare. Almost 50% of the patients continued the medication during the third trimester of pregnancy and a third of them until the time of delivery, and there was no increased risk of neonatal infection or infections during the first year of life [133]. Live vaccines are contraindicated in the first six months of life, especially BCG vaccine (Bacillus Calmette–Guérin) [134].

Initially used to compensate for the deficient immune response in immunocompromised patients, iv IgG is now a widely used therapy in autoimmune and systemic inflammatory diseases [135,136]. Iv IgG is obtained from plasma of healthy blood donors and also has applicability in a wide range of pathologies associated with pregnancy. Its clinical utility comes from its ability to interfere with FcRn. Large quantities of exogenous IgG determine saturation of FcRn and as a result, the excess of pathological IgG that could not bind to FcRn will be destroyed. Consequently, there will be lower levels of pathological maternal antibodies transferred to the fetus [12,137].

Preventing harmful antibodies from reaching the fetal circulation, iv IgG is currently used in autoimmune thrombocytopenia, myasthenia gravis and systemic lupus erythematosus [138]. Also, iv IgG represents the first line of therapy in preventing recurrence of neonatal alloimmune thrombocytopenia, initiated early in the first or second trimester of pregnancy [139,140]. Hyperimmune intravenous immunoglobulin is the subject of investigation for preventing congenital cytomegalovirus infection in women with primary infection in the first trimester of pregnancy. The results of the studies undertaken so far are conflicting, and at the moment there is no clear recommendation on routine iv IgG therapy for primary maternal cytomegalovirus infection [141,142].

## 6. Conclusions

The transplacental passage of maternal IgG antibodies is of great importance to the fetus and newborn, offering suitable protection until maturation of the immune system and until active immunization through vaccination is allowed. On the other hand, in some conditions such as maternal autoimmune disorders or maternal immune responses against paternally inherited fetal antigens, the physiological passage of immunoglobulin can have a deleterious effect upon the fetus to various degrees of severity and with potential long-term implications.

## Figures and Tables

**Table 1:** Maternal and fetal laboratory changes in different pathologies.

Autoimmune Disorders			Alloimmune Disorders	
Maternal	Fetal		Maternal	Fetal
Anemia			Anemia	
↓Hb ↑ Bilirubin ↑ LDH	↓ Hb		Normal Hb	↓ Hb
NThrombocytopenia			Thrombocytopenia	
↓ PLT	↓ PLT		Normal PLT	↓ PLT
Graves’ disease			Hepatitis/Neonatal hemochromatosis	
↓ THS ↑ fT4		Hyperthyroidism ↓ THS, ↑ fT4 or Hypothyroidism ↑ TSH, ↓ fT4	Normal Alt/Ast	↑ Alt/Ast Hyperferritinemia ↑ Transferrin saturation ↓ Prothrombine time

Hb = hemoglobin, LDH = lactate dehydrogenase, PLT = platelets, TSH = thyroid-stimulating hormone, fT4 = free thyroxine, Alt = alanine transferase, Ast = aspartate transferase; ↑—increase; ↓—decrease.
